# Assessment of the Psychosocial Care Center multidisciplinary team from users’ and family members’ perspective

**DOI:** 10.1590/0034-7167-2022-0645

**Published:** 2023-08-21

**Authors:** Jéssica dos Santos Pini, Paula Antunes Bezerra Nacamura, Camila Harmuch, Maria Antonia Ramos Costa, Bianca Cristina Ciccone Giacon-Arruda, Cremilde Aparecida Trindade Radovanovic, Maria Aparecida Salci, Marcelle Paiano

**Affiliations:** I Universidade Estadual de Maringá. Maringá, Paraná, Brazil.; II Universidade Estadual do Paraná. Paranavaí, Paraná, Brazil; III Universidade Federal do Mato Grosso do Sul. Campo Grande, Mato Grosso do Sul, Brazil

**Keywords:** Health Services Evaluation, Mental Health Services, Patient Care Team, Professional-Family Relations, Mental Disorders, Evaluación en Salud, Servicios de Salud Mental, Grupo de Atención al Paciente, Relaciones Profesional-Familia, Trastornos Mentales, Avaliação em Saúde, Serviços de Saúde Mental, Equipe de Assistência ao Paciente, Relações Profissional-Família, Transtornos Mentais

## Abstract

**Objectives:**

to assess the multidisciplinary team of a Psychosocial Care Center I from users’ and family members’ perspective.

**Methods:**

an evaluative study, anchored in the fourth generation evaluation theoretical-methodological framework, carried out in a Psychosocial Care Center I, from September 2021 to March 2022. Eleven users and 06 family members participated. Data were collected through non-participant observation, individual interviews and negotiation sessions, and analyzed using the Constant Comparative Method, using the MAXQDA software.

**Results:**

the team develops its care based on individual and collective care, with integrated and complementary work by professionals. They seek to facilitate treatment initiation and continuation, considering health needs and offering support, understanding and guidance to users and their families.

**Final Considerations:**

the multidisciplinary team’s work is based on the psychosocial paradigm, which can qualify care and strengthen the service role in the mental health network.

## RESUMO

**Objetivos:**

avaliar a equipe multiprofissional de um Centro de Atenção Psicossocial I na perspectiva dos usuários e familiares.

**Métodos:**

estudo avaliativo, ancorado no referencial teórico-metodológico da avaliação de quarta geração, realizado em um Centro de Atenção Psicossocial I, de setembro de 2021 a março de 2022. Participaram 11 usuários e 06 familiares. Os dados foram coletados por observação não participante, entrevistas individuais e sessões de negociação, e analisados pelo Método Comparativo Constante, com uso do software MAXQDA.

**Resultados:**

a equipe desenvolve sua assistência pautada em atendimentos individuais e coletivos, com trabalho integrado e complementar dos profissionais. Busca facilitar o início e a continuidade do tratamento, considerando as necessidades de saúde e ofertando apoio, compreensão e orientações ao usuário e familiares.

**Considerações Finais:**

a atuação da equipe multiprofissional se pauta no paradigma psicossocial, o que pode qualificar o cuidado e fortalecer o papel do serviço na rede de saúde mental.

## RESUMEN

**Objetivos:**

evaluar el equipo multidisciplinario de un Centro de Atención Psicosocial I desde la perspectiva de los usuarios y familiares.

**Métodos:**

estudio evaluativo, anclado en el referencial teórico-metodológico de la evaluación de cuarta generación, realizado en un Centro de Atención Psicosocial I, de septiembre de 2021 a marzo de 2022. Participaron 11 usuarios y 06 familiares. Los datos fueron recolectados a través de observación no participante, entrevistas individuales y sesiones de negociación, y analizados mediante el Método Comparativo Constante, utilizando el software MAXQDA.

**Resultados:**

el equipo desarrolla su asistencia a partir de la asistencia individual y colectiva, con trabajo integrado y complementario de los profesionales. Busca facilitar el inicio y la continuación del tratamiento, considerando las necesidades de salud y ofreciendo apoyo, comprensión y orientación a los usuarios y sus familias.

**Consideraciones Finales:**

la actuación del equipo multidisciplinario se basa en el paradigma psicosocial, que puede calificar la atención y fortalecer el papel del servicio en la red de salud mental.

## INTRODUCTION

The Psychosocial Care Center (CAPS – *Centro de Atenção Psicossocial*) is the main specialized care service, which includes comprehensive care and articulation with the Psychosocial Care Network’s care points^(1)^. It was regulated, initially, by Ministerial Ordinance 336, in 2002, and by Consolidation Ordinance 3, of 2017, which enabled its expansion and recognition of its relevance in health care^(1, 2, 3)^.

According to Consolidation Ordinance 3, CAPS consists of a community-based and territorial service intended for the clinical treatment of individuals with suffering or severe and persistent mental disorder, including those with needs arising from the use of alcohol and/or other drugs, through care and rehabilitation actions^(1)^. In this regard, for the inclusion of these individuals in society, it is necessary to stabilize mental disorders so that they do not require hospital admissions, and individual and collective care is carried out by a multidisciplinary team, based on interdisciplinary care^(1, 4)^.

The multidisciplinary team can be constituted in different ways and have different professional categories, as long as the minimum parameters required in ordinances that regulate the CAPS are respected, according to their modality. The team, as a whole, is involved in user and family care from their arrival at the service until discharge^(1)^.

The integration of professionals’ knowledge, actions and speeches, who must work together, is one of the tools to guarantee the excellence of the care offered^(5)^. Each professional brings to their practice the knowledge of their technical training and, with team members, manages to go beyond what they would do alone, permeating the user’s and the family’s needs^(6)^.

According to the psychosocial paradigm, users and their families are subjects of mental health care and are co-responsible for its success, together with health professionals. The relationship between them should aim to welcome, support and meet health needs, in order to contribute to home care and social inclusion of users. Thus, the CAPS team needs, in its daily actions, to bring the family closer to the service and user care^(7)^.

The demands, concerns and processes arising from the new way of caring for mental health require a deepening in relation to multidisciplinary CAPS team’s work. This becomes essential in a CAPS I, which has the smallest team, in relation to the service’s other modalities, and must assist a wide audience, composed of children, young people and adults with different needs resulting from mental disorders and/or alcohol use^(1)^.

For this, it is possible to have evaluative research that, through an assessment process, has the final objective of qualifying the care provided in the service^(8)^. Among the qualitative assessments used in health services, the fourth generation evaluation (FGE) stands out, whose theoretical-methodological framework is anchored in the constructivist paradigm and in the hermeneutic-dialectical process. Participants’ claims, concerns and issues (CCI) determine what should be addressed in this assessment, making it responsive^(9)^.

In the FGE carried out at CAPS, service users and their families are considered groups of interest in the evaluative research, since they are impacted by the care offered by the multidisciplinary team or by the assessment itself and, therefore, must participate in the assessment process. By involving different groups present in the service’s daily life, greater reliability of results is guaranteed, since it includes the actors of the care process, according to health care assumptions^(9)^.

Given the above, it is understood that revealing multidisciplinary CAPS team’s work, according to the assessment of service users and their families, is necessary to establish interventions that contribute to qualifying mental health care, which justifies carrying out this evaluative research, developed to answer the following question: how do CAPS users and their families assess the service’s multidisciplinary team?

## OBJECTIVES

To assess the multidisciplinary team of a CAPS I from service users’ and their families’ perspective.

## METHODS

### Ethical aspects

The study was conducted in accordance with national and international ethics guidelines, and was approved by the Research Ethics Committee of the *Universidade Estadual de Maringá*. After being clarified about the research, all individuals involved in the study signed the Informed Consent Form. To maintain participant anonymity, their names were replaced by the letter “U”, for service users, or “F”, for family members, followed by a sequential number corresponding to the order of interviews in each group of participants.

### Study design

This is a case study^(10)^ with a qualitative, evaluative approach, anchored in the FGE methodological theoretical framework^(9)^, guided by the Consolidated criteria for REporting Qualitative research (COREQ) precepts.

### Study setting

The study took place in a CAPS I in a small town in the state of Paraná. As it is the only CAPS in the municipality, and according to its modality, it assists individuals in mental suffering and/or use of alcohol and other drugs, including children and adolescents, working from Monday to Friday, from 7:30 am to 5:00 pm.

It assists, on average, 380 users per month. It offers consultations, individual follow-up, groups and therapeutic workshops, and home visits for users and their families, in addition to welcoming spontaneous demands and referrals made by other health services. Its multidisciplinary team is made up of 13 professionals, such as psychiatrists, psychologists, nurses, social workers, social educators, artisans, occupational therapists and service coordinators.

The choice of CAPS I for developing the research is justified by the fact that it is the most implemented service modality in the country and that must meet all the general population’s mental health needs, including those resulting from the use of alcohol and/or other drugs, having the smallest minimum team required by ordinance, compared to the other modalities^(1, 2)^. Furthermore, the aforementioned service is located in a municipality where the researcher worked professionally, prior to the development of this research, a period in which he had contact with CAPS users and their families.

### Data source

A total of 11 users and 6 family members participated in the study. We included users if they were over 18 years old, had received care at the service in the last three months and were indicated by the Hermeneutic-Dialectic Circle (HDC) as the next respondent. We included family members of users assisted at the service in the last three months, over 18 years old, residing with users and being indicated by HDC as the next respondent.

For both interest groups, we excluded those who were in crisis at the time of data collection and have cognitive impairment for study participation, identified from the Mini Mental State Examination, translated and adapted, which was applied by the researcher^(11)^. In this study, there was no refusal, withdrawal or exclusion among the individuals invited to participate.

### Data collection and organization

Data collection took place from September 2021 to March 2022, being carried out by the main researcher. First, contact was made with the service, for presentation of research and agreement for the researcher to remain in the field. From this, non-participant observation began on site, on all days and periods of care, totaling 95 hours.

A field script was used to conduct the observation, containing aspects of structure, resources, routine and processes to be considered for in-depth knowledge of the service, and a field diary for record, comments and reflections on what was observed in the insertion of the researcher. This step made it possible to know and monitor the various services provided at CAPS, as well as existing flows and dynamics, in addition to promoting coexistence with users.

Afterwards, the stage of semi-structured individual interviews was carried out. For presentation of the research and invitation to participate, users were approached individually at the end of the CAPS service. Reapproaching with the family members took place during a home visit with the service team, since collective services for them, such as meetings and family groups, were suspended due to restrictions imposed by the COVID-19 pandemic.

At this first moment, a date and time were scheduled for the interview, which took place at the service, in the case of users, or at their own residence, in the case of family members, always in a place that guarantees privacy and confidentiality and only with the presence of the researcher.

HDC was used as a data collection method for each interest group. Group participants (users and family members) were selected by convenience. Users’ HDC was started with one of the individuals who has been monitoring the service since its implementation. Family members’ HDC, on the other hand, started with a family considered to have a great participation in users’ treatment, according to the multidisciplinary team. The interviews were conducted with the initial guiding questions: how is the CAPS service? What are the service’s strengths and weaknesses?

For HDC continuity, the respondent indicated the next participant. With the analysis of his interview, the referent construction was obtained, and, based on it, other questions were incorporated into the interview script for the next participant. This process occurred until the last interview, and participants answered, in addition to the initial questions, those arising from the analysis of previous interviews and their constructions. The elaborate constructions are shown in Chart 1.

**Chart 1:** Hermeneutic-Dialectic Circle constructions by Psychosocial Care Center users and family members

	Triggering questions	Buildings elaborated at the end of HDC
Users	How is the service at the CAPS you attend? What are the CAPS’ strengths and weaknesses?	- Welcoming and access to the service. - Individual and collective care. - Professionals’ and team’s work. - Care in crisis situations. - Actions for treatment compliance. - Quality of care offered. - Comprehensive care. - Socialization and social reintegration. - Family care.
Family member	How is the CAPS service that your family member attends? What are the CAPS’ strengths and weaknesses?	- Family participation in the service. - Treatment access and compliance. - Family care. - Service features. - Professional and team role. - Team as a support for crisis and uncertainties. - Team responsibility in the success of outpatient treatment.

*HDC – Hermeneutic-Dialectic Circle; CAPS – Psychosocial Care Center.*

Data collection was discontinued when there was redundancy in the information obtained or when it fit into two or more conflicting constructions^(9)^. Thus, the HDC of users was completed with 11 participants, and that of family members, with 6 participants, and no family member and user belonged to the same family.

Each interview lasted, on average, 43 minutes: the shortest lasting 10 minutes and the longest lasting 2 hours and 3 minutes. All data collection steps were performed by the main researcher. The interviews were audio-recorded with the support of a smartphone, after participants’ consent, and transcribed, with adaptations, to remove language defects and correct speech errors, without interfering with content and meaning.

### Negotiation session

At the end of HDC, a negotiation session was scheduled with each group of participants, separately, with invitation made by telephone call. During negotiation, participants had access to CCI and constructions that emerged from the interviews, which were synthesized, organized and presented with the support of a data show. Printed content was provided for each participant.

With this, the group can discuss and clarify the constructions and decide what would remain as a result of the study, according to participants’ agreement. It should be noted that there is no minimum number of participants for the negotiation to be considered valid.

User negotiation had the participation of eight people, lasting 1 hour and 55 minutes. None of the 3 missing users explained their absence. The topics for user validity were: actions that guarantee outpatient treatment; quality of service; the impact of the care offered; service features; care comprehensiveness; the CAPS and the Psychosocial Care Network; family care; structure and ambience; and social control.

In family member negotiation, four families were present, who appreciated and discussed the following topics: treatment access and compliance at the service; outcome of treatment at CAPS; service features; family in CAPS; and physical structure. This session lasted 1 hour and 27 minutes, and the two absent family members did not inform the reason for their non-attendance.

### Data analysis

For data analysis, the Constant Comparative Method was used, which allows analyzing data during collection. Each interview was analyzed before the next one, enabling performing the HDC and the joint construction of the research result, inherent to FGE^(9)^.

It started with the identification of information units, which are relevant paragraphs or sentences. These were coded, grouped, when related to the same content, and gave rise to provisional categories. From the negotiation with participants, the definitive categories of this study were constructed^(9)^. In addition to the interviews, the information contained in a field diary and the researcher’s other observations were also considered in the analysis.

The MAXQDA^(12)^ software was used to organize and present the data, considering the occurrences/recurrences of terms and expressions of analyzed interviews. With this software, it was possible to build a cloud of words related to the information units, providing a visual display of the frequency of words.

## RESULTS

Users who participated in the study are aged between 34 and 67 years old, are followed up at CAPS for at least two years and three of them have been undergoing treatment at the service since the beginning of its operation: 14 years ago. There was a predominance of women and users with a single diagnosis of mental and behavioral disorder. Present diagnoses are depressive disorder, bipolar affective disorder, schizophrenia, mental and behavioral disorder due to alcohol use, and mental and behavioral disorder due to cannabinoid use.

Users attend CAPS weekly to participate in collective activities, with nine participating in therapeutic workshops and two participating in a therapeutic group for users of psychoactive substances, both with weekly frequency and duration of two hours. All are seen by the psychiatrist, on average every 90 days, and have already received psychotherapeutic care, and only two still have sporadic consultations with the psychologist. Three users have also received individual care with the occupational therapist.

Family members have an average age of 62 years, ranging from 25 to 79 years, and are the mother, father, brother, son or spouse of individuals who attend CAPS. Accompany their family members to medical appointments and crisis care, participating in meetings for families, offered sporadically. The time of participation in CAPS, as a family, is from 04 to 12 years, and their relatives monitored in the service have diagnoses of schizophrenia, depression and use of psychoactive substances. They attend collective activities weekly, have periodic psychiatric consultations and none is in individual care with another professional on the team.

**Figure 1:**
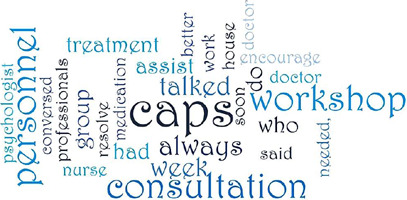
Word cloud created by the authors based on research data in the MAXQDA software, Brazil, 2022

The assessments carried out by participants of this study originated the word cloud presented in Figure 1, with the organization of the information obtained from its occurrence and recurrence in speeches.

In line with the constant comparative method, it was possible to construct two categories. The first is “Importance of the multidisciplinary team for optimization of care”, with the following words “consultation”, “workshop” and those referring to professional categories such as “nurse”, “doctor” and “psychologist” highlighted. In the second, “The multidisciplinary team as a treatment facilitator”, the words “do”, “converse”, “serve” and “resolve” stood out”.

### Importance of the multidisciplinary team for optimization of care

In this category, it is possible to observe, according to family members’ and users’ perceptions, the actions carried out by professionals who make up the multidisciplinary team. In the eyes of the family member, the closest professionals are those who work in welcoming user/family in the service (welcoming) and in medical consultations (both routine and during a crisis).

During the period of non-participant observation at CAPS, it was identified that many families attend CAPS, especially in those moments mentioned above, precisely because it is a moment of crisis and treatment that requires the presence of the family.

*Of the people who work there, I only know the doctor, who monitors the appointments, and there are people who assist us when we arrive at the CAPS, who are at the welcoming.* (F6)

*We always accompany him when he is not well, to tell him what happened, how the treatment is going and see what the doctor will decide. Therefore, I know the doctor better and who assists me when I get there.* (F2)

In addition to the medical consultation and welcoming at the welcoming, users highlighted the work of professionals in workshops and therapeutic groups.

*In the workshop I come to, there are artisans. They are very close to those who participate, because, in addition to teaching how to make handicrafts, they talk a lot with us.* (U3)

*The therapist leads the therapeutic group, for those who use some substance. He accompanies and supports us*. (U10)

Other professionals were also mentioned by family members and users, such as the occupational therapist, psychologist and nurse, demonstrating that the care offered by them must be added to the actions of all other team members.

*The occupational therapist accompanied my daughter at the beginning, until she stabilized and started the workshop. The psychologist too. It was very important when I quit drinking.* (F3)

*There’s a nurse here. She sees me on the day of the consultation, to weigh and check the* [blood] *pressure. When I need something with the doctor, I come and talk to her, so she can see how to solve it.* (U4)

It is observed that the interviewees perceive the team as integrated and articulated, because, even though it is composed of professionals with different backgrounds, they try to be in tune and work together to solve the different situations of daily life.

*Each person who works there has their role, but everyone is organized to do what is necessary and find a solution. Each one brings what they know, treatment is very good.* (F1)

*I know that if I get sick and come here, whoever is here will take care of me. It doesn’t matter her profession, she will receive me, listen to me and try to solve it. They take good care of us, talk and act in the best way.* (U7)

Therefore, family members and users understand that actions are carried out by professionals in search of comprehensive mental health care, emphasizing the importance of all members as responsible for care.

### The multidisciplinary team as a treatment facilitator

The multidisciplinary team is assessed as an instrument for CAPS processes to take place and allow treatment to occur in the best possible way, guaranteeing adequate care to service users and their families.

In this sense, the receptiveness to start and maintain the treatment was cited by family members and users as one of the multidisciplinary team’s strengths.

*Since we found out that he wasn’t well, that we went after him, they immediately took care of him. The CAPS people talked to him and they scheduled to start.* (F2)

*I went in a group, to stop smoking, with the CAPS nurse. She saw me full of symptoms and made an appointment with the psychologist here. When I came, professionals assessed me and said that I needed to be treated.* (U7)

Furthermore, professionals mobilize so that the treatment offered by the service is the most appropriate to users’ situation, ensuring that their health needs are met.

*The professionals there will fix it if she doesn’t feel well. Even if the consultation is not close, they talk to each other, discuss with a doctor or psychologist. If necessary, pass it on to the consultation. They get a prescription, if the medicine is running out.* (F3)

*When I needed a certificate from the INSS, I spoke to the CAPS and they resolved it, even without having an appointment with a doctor that week.* (U5)

The support and guidance offered by the team, especially to family members, were cited as reinforcement for the best treatment, especially in difficult situations.

*When something more serious happened, they came to the house to help solve it. They talk to him, assess the case and decide with us how to do it.* (F4)

*There’s a friend here whose family didn’t help much with treatment, so the CAPS called, talked, explained, and they completely changed. Today, they encourage you to come.* (U6)

Understanding and respect are also present in multidisciplinary team’s work, guaranteeing the autonomy of users and the choice of the family in conducting treatment.

*When we arrive at the workshop, they ask what we want to do. Each one can choose. If you don’t want to do it, no one is obligated. They invite and encourage, but let you decide.* (U9)

*In the therapeutic group, those who cannot stop using substances, they do not blame. They say to continue participating and to try to stop, which is better.* (U10)

*I said that I no longer wanted her to dance at activities here, and they accepted. They talked to me, we decided and she continued to participate.* (F6)

## DISCUSSION

In this study, it was evidenced that medical consultations and the welcome given at the service welcoming were pointed out by participants as important professional actions carried out, as these are the moments in which there is greater participation of family members.

It is possible to observe that, when the family does not have contact with the rest of the team, there is distancing and lack of knowledge of other professionals who make up the service. Therefore, it is necessary that all professionals include it, along with users, in the care process^(7)^. Research carried out with mental health managers showed that family participation becomes a source of information so that treatment is better directed^(13)^, explaining the importance of its linkage to the entire health team.

Despite the importance given to medical consultations, corroborated by the term “consultation” in the word cloud, in our study, other professionals were also cited, with emphasis on those who carry out collective activities, as demonstrated by the terms “workshop” and “groups”.

This finding should be highlighted, as it demonstrates the transition to the psychosocial care model, considered ideal by Consolidation Ordinance 3, which defines the multidisciplinary team for care at CAPS^(1)^. All professionals must interact with each other and with users, in an interdisciplinary and interprofessional way, to provide care in its entirety. However, there are many difficulties in carrying out this transition, especially with regard to the importance given to medicalization and medical care^(14, 15)^.

The effectiveness of the psychosocial model determines the need for multidisciplinary work, in which each category uses the specific knowledge of its training to develop its actions^(16)^. Participants highlighted that this has been happening in the service, demonstrating that each professional acts in different moments of care, individual or collective.

Although multidisciplinary work provides a view from different angles, it must be part of a single care plan for conducting mental health treatment: the Singular Therapeutic Project^(1)^. If the different types of knowledge are combined in a common objective, it is possible to go further and guarantee psychosocial care. In mental health services, this is essential, as existing demands are not exhausted by just one professional category^(17)^.

When these professionals come together to develop joint work, which mixes the different knowledge and practices in search of comprehensive care, there is an interprofessional action^(16)^. This is present in participants’ speeches, who identify the work of certain professionals integrated with others and even mention team’s work as a whole.

It is known that interprofessional work still faces challenges for it to be effective in mental health services and that, notably, it coexists with multidisciplinary work, being responsible for different moments of health care that complement each other^(18)^.

The results of this study suggest that there is union and a good relationship between the different professionals, which directly reflects on teamwork and on the care offered, which was proven in the service observation. For proper teamwork to take place, interpersonal relationships must be healthy and constructive, which can be achieved when the work process is strengthened and permeated by conversations, negotiations and the construction of routines^(19)^.

Unlike what was found, it is common for CAPS evaluative studies to demonstrate that the team’s work has weaknesses related to the work process^(20, 21, 22)^, directly impacting interpersonal relationships. It is believed that in the assessed service, as it is a CAPS I, the interprofessional relationship is facilitated, since it has a smaller team and is located in a small city^(1)^, where many know each other and live together outside the work environment.

As a consequence of the good relationship between professionals, division of work, participation in discussions, mutual help to solve problems and communication between them and with management is facilitated. Thus, it is possible to strengthen teamwork and organize their process^(18)^.

It was possible to observe in the results that professionals are organized to guarantee the beginning of users’ treatment and its continuity, developing actions that contribute to service compliance. It is known that team collaboration, as a whole, and the understanding that each one is important to welcome and support treatment, are essential to qualify this moment^(23)^.

The assessed team strives to meet users’ health needs. Situations presented by them, by their families, or observed by professionals, should be motivation for the team to seek solutions that match the reality experienced, ensuring expanded care^(24)^ that considers individuals’ physical, emotional, social, family and spiritual aspects^(25)^.

Moreover, in the quest to maintain treatment and reduce crises, the CAPS team offers adequate support and guidance to the family, through groups, consultations and activities that include family members in care, to be co-responsible for therapy compliance and success. This support helps to reduce family members’ suffering and emotional burden, bringing them closer to their loved ones with mental disorders and the care they receive at the service^(7)^.

A study in a CAPS AD showed that users and families positively assess the guidelines offered by the team, highlighting the quality of information and the direction they receive^(15)^. In a child-juvenile CAPS, guidance to the family is understood as a facilitator of communication between the family and the team, when carried out with clarity and coherence with the demands^(26)^. By offering enough knowledge to the family, they are able to make decisions about users’ treatment without putting them at risk, since their capacity for analysis and intervention is expanded. In this context, it is up to professionals to support family autonomy and empowerment^(27)^.

In this study, it was possible to observe the respect for the family’s decisions by the service team, even when they do not effectively result from situations related to the mental disorder. Users are also heard in their wishes, providing autonomy development and exercise, which is one of the objectives of CAPS. At the same time that the team provides the emancipation of users inside and outside the service, it also monitors and assesses decisions, as it must consider their will without failing to assist them properly^(28)^.

Finally, it is consistent to say that the assessed team has approached psychosocial care, as it seeks to alleviate the remnants of the period prior to the Psychiatric Reform, through multidisci-plinary action, interprofessionality, subjects’ autonomy and users’ observance as protagonists of their treatment and their lives. Thus, the psychosocial paradigm precepts are strengthened in professional practices, as recommended^(1)^.

### Study limitations

One of the limitations of this study is its work in only one mental health service, which may restrict the results to the local context. Furthermore, it is understood that the fact that this assessment does not cover professionals’ view may limit it, since these are also part of the service’s interest groups. It is believed that, from professionals’ perspective in relation to their actions, other results may emerge, complementing those found in this study.

### Contributions to nursing, health, and public policies

With this assessment, it was possible to observe that the multidisciplinary CAPS team develops its actions according to the premises of psychosocial care and deinstitutionalization. This contributes to strengthening CAPS in the mental health and community and territorial care network. Therefore, public policies need to be guaranteed as well as funding for the implementation and expansion of mental health services.

Specifically for nursing, the results demonstrate their work integrated with the care of other professionals as a member of the multidisciplinary team, but without individual relevance in the mental health care process. Thus, it is up to professionals in this category to identify their practices and redirect them so that it is possible to occupy their primary place in care, strengthening the science of nursing.

## FINAL CONSIDERATIONS

The results of assessment show that the CAPS team develops its care based on the psychosocial paradigm, in which individual and collective actions complete each other, being thought and organized by the team as a whole, with joint and complementary work being carried out. In addition to this, it takes the position of facilitating treatment, considering health needs and offering support and guidance to users and their families. This qualified care strengthens CAPS role as a community and territorial service of the mental health network.

However, we should reflect on discreet family participation, which happens mainly in crisis care and medical consultations. Family involvement is paramount for treatment, stabilization of users’ clinical condition and resocialization, and therefore, the team needs to rethink the attention given to it, making frequent and diversified visits.

Finally, it is necessary to consider that participants assessed the team’s work, pointing out, as a priority, its potential. This may indicate that professionals are resolute in the face of problems that arise or that users and their families tend to minimize existing problems in the service. Faced with this possibility, it is necessary for the CAPS team to equip users and their families to understand their rights and encourage participation in the service’s social control, which will contribute to successful and qualified care.

## Data Availability

https://doi.org/10.48331/scielodata.V8GDBY
